# Validation of Reference Genes for Expression Studies during Craniofacial Development in Arctic Charr

**DOI:** 10.1371/journal.pone.0066389

**Published:** 2013-06-13

**Authors:** Ehsan Pashay Ahi, Jóhannes Guðbrandsson, Kalina H. Kapralova, Sigríður R. Franzdóttir, Sigurður S. Snorrason, Valerie H. Maier, Zophonías O. Jónsson

**Affiliations:** 1 Institute of Life- and Environmental Sciences, University of Iceland, Reykjavik, Iceland; 2 Biomedical Centre, University of Iceland, Reykjavik, Iceland; Beijing Institute of Genomics, Chinese Academy of Sciences, China

## Abstract

Arctic charr (*Salvelinus alpinus*) is a highly polymorphic species and in Lake Thingvallavatn, Iceland, four phenotypic morphs have evolved. These differences in morphology, especially in craniofacial structures are already apparent during embryonic development, indicating that genes important in the formation of the craniofacial features are expressed differentially between the morphs. In order to generate tools to examine these expression differences in Arctic charr, the aim of the present study was to identify reference genes for quantitative real-time PCR (qPCR). The specific aim was to select reference genes which are able to detect very small expression differences among different morphs. We selected twelve candidate reference genes from the literature, identified corresponding charr sequences using data derived from transcriptome sequencing (RNA-seq) and examined their expression using qPCR. Many of the candidate reference genes were found to be stably expressed, yet their quality-rank as reference genes varied considerably depending on the type of analysis used. In addition to commonly used software for reference gene validation, we used classical statistics to evaluate expression profiles avoiding a bias for reference genes with similar expression patterns (co-regulation). Based on these analyses we chose three reference genes, ACTB, UB2L3 and IF5A1 for further evaluation. Their consistency was assessed in an expression study of three known craniofacially expressed genes, sparc (or osteonectin), matrix metalloprotease 2 (mmp2) and sox9 (sex-determining region Y box 9 protein) using qPCR in embryo heads derived from four charr groups at three developmental time points. The three reference genes were found to be very suitable for studying expression differences between the morphotypes, enabling robust detection of small relative expression changes during charr development. Further, the results showed that sparc and mmp2 are differentially expressed in embryos of different Arctic charr morphotypes.

## Introduction

The head of teleost fish, particularly their trophic apparatus, contains many movable elements which make it one of the most complex and integrated musculo-skeletal systems in vertebrates [1]. The development of these elements requires complicated interactions between derivatives of all three germ layers in setting up and tuning the relevant molecular pathways [2]. Fish exhibit tremendous functional diversity in their craniofacial skeleton and provide an interesting model for studying evolution of those features using developmental genetics. Phenotypic variation in the shape and formation of the trophic apparatus among related species has been most extensively studied in cichlids and zebrafish [1], [3], [4]. Intraspecies comparisons are also of great interest here, especially in systems where phenotypically distinct morphs have evolved [5]–[7]. Arctic charr (*Salvelinus alpinus*) is amongst the most thoroughly studied systems of polymorphism in fish [8]. In Lake Thingvallavatn in Iceland four residential morphs of Arctic charr are found, large benthivorous (LB), small benthivorous (SB), a pelagic planktivorous (PL) and piscivorous (PI) charr [9]. The morphs differ in diet, morphology, behaviour and life history characteristics [9]–[12]. The two benthivorous morphs, feeding largely on snails, have an overshot mouth (a benthic morphotype), while the pelagic morph, feeding mainly on zooplankton, and the piscivorous morph have a terminal mouth (a pelagic morphotype) [11]. The adaptive nature of morph formation among the Arctic charr of Lake Thingvallavatn has been demonstrated in a series of laboratory rearing experiments [13]–[15]. These studies show a strong genetic component with a significant maternal effect on the development of trophic morphology and feeding behaviour. On a population level, recent studies of 10 microsatellite markers in the two most abundant morphs have demonstrated low, but significant, genetic differentiation between these morphs, consistent with strong reproductive isolation throughout the Holocene [16].

Heterochrony is thought to be an important mechanism in the evolution of the morphs illustrated in a study where bones in the small benthivorous morph were shown to start ossifying earlier and/or faster than in the pelagic morph [15]. The low level of genetic differentiation amongst the morphs, but distinct phenotypic differences suggests a mechanism based on a few regulatory factors operating early in the development of key trophic traits. To date little is known about the expression of such regulatory elements in charr. Differences in temporal expression of the transcription factor Pax 7 between SB and LB morphs have been observed [17], but large scale comparisons at the expression level are lacking.

In recent years high-throughput transcriptome sequencing (RNA-Seq) has emerged as a way to profile gene expression [18] and can be used as a powerful tool to contrast expression for example in the pelagic and benthic morphotypes. The method has clear advantages. There is no need for probes designed from previously-known transcripts, novel transcripts can be detected in organisms without a sequenced genome, such as Arctic charr, and expression levels can be quantified [18]. Expression profiles from RNA-Seq data can be validated using PCR based approaches [19], [20]. Quantitative real-time PCR (qPCR) is a widely used method to study gene expression and a cost effective way to examine expression patterns of key candidate genes, e.g. genes identified from RNA-seq developmental profiling. Measuring and comparing expression levels of genes of interest requires normalisation against the expression levels of reference genes [21], [22]. Some of the classical reference genes, e.g. the genes encoding ACTB, GAPDH, EF1α and ribosomal proteins, have been examined in fish [23]–[29]. There is however general agreement that no perfect reference gene exists for assessing differential expression levels at various developmental stages in different tissues, body compartments and organisms [30]. Therefore the validation of reference genes under defined experimental conditions or at defined developmental stages is crucial [31], [32].

The aim of this study was to identify and validate stable reference genes for quantitative real-time PCR during craniofacial development of Arctic charr embryos. To this end we selected a number of potential reference genes originating from independent pathways and based on previous studies in other fish species [23]–[29]. We used published sequences of these genes and transcriptome sequencing data obtained from Arctic charr embryos to design primers for quantitative real-time PCR. Candidate reference genes were analysed in a number of ways; (i) by testing for stability in expression levels among developmental time points and Arctic charr groups, (ii) by testing for consensus between expression levels derived from qPCR and RNA-seq data, and (iii) by testing consistency of results, when used to normalise expression levels of three developmental genes.

## Methods

### Sampling of parents and setting up of embryo groups

**Figure 1:**
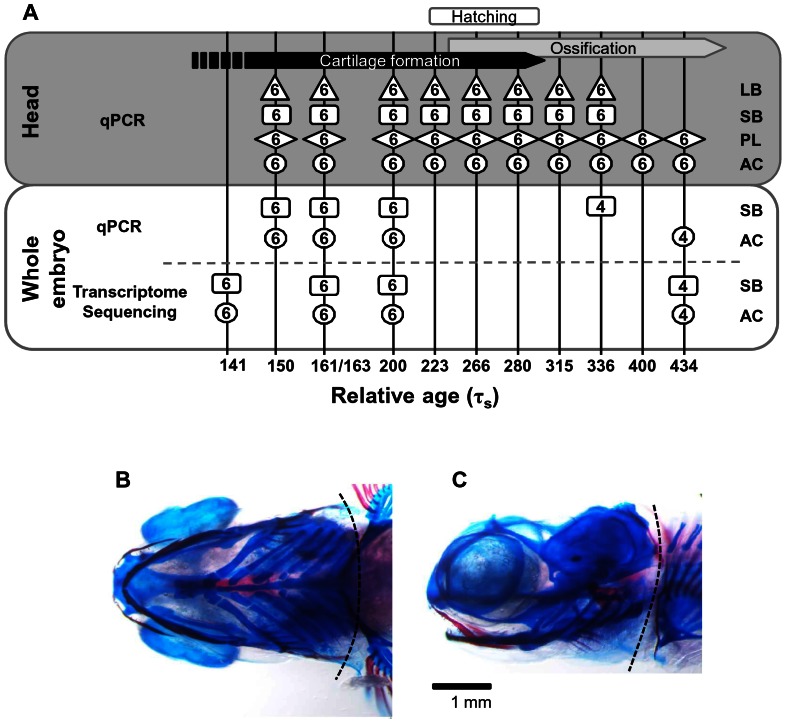
A scheme of sampling and analyses of Arctic charr development. (**A**) Embryos were collected at the indicated relative age (represented by vertical lines). Either whole embryos or heads of the indicated charr groups were used for RNA extraction (LB: large benthivorous charr; SB: small benthivorous charr; PL: planktivorous charr; AC: aquaculture charr). The numbers (in boxes, circles and diamonds) indicate the number of individuals pooled for each extraction. The RNA was reverse-transcribed and the cDNA used for qPCR or transcriptome sequencing as shown. Bars at top display approximate time points of cartilage formation, ossification and hatching (unpublished data). (**B–C**) Ventral and lateral views of a planktivorous head at 336 τ_s_ with a dashed line representing the decapitation line in front of the pectoral fin for head sample collection. Embryos were stained with alcian blue (for cartilage) and alizarin red (for bone) according to the described method [74] with some alterations.

The embryo series come from four parental groups sampled in two years. In 2009 embryos from the Holar aquaculture stock (AC) and the small benthivorous charr (SB) from Lake Thingvallavatn were collected. In 2010 embryos from AC and SB were collected as well as the small planktivorous (PL) and the large benthivorous (LB) charr from Lake Thingvallavatn. Fishing permissions were obtained from the Thingvellir National Park Commission and the owner of the farm Mjóanes. Fish were killed by a sharp blow to the head and for each group, eggs from several females were pooled and fertilized using milt from several males from the same group. Eggs were reared at approximately 5°C in a hatching tray (EWOS, Norway) under constant water flow and in complete darkness at the Holar University College experimental facilities in Verið, Sauðárkrókur. The water temperature was recorded twice daily and the average was used to estimate the relative age of the embryos using tau-somite (τ_s_) units defined as the time it takes for one somite pair to form at a given temperature [33]. Embryos were collected directly into RNA-later solution (Ambion) at the indicated relative age (Figure 1) and stored at −20°C until further use. The rearing and collection of the embryos was performed according to Icelandic regulations (licence granted to Holar University College aquaculture and experimental facilities in Verið, Sauðárkrókur).

### RNA extraction and cDNA synthesis

Embryos were dechorionated under the light microscope (Leica S6E) and the yolk was discarded. Embryos sampled in 2009 were used for transcriptome sequencing. SB and AC whole embryos, at the relative age indicated in Figure 1A (lower panel, sequencing), were homogenized with a disposable Kontes Pellet Pestle Cordless Motor tissue grinder (Kimble Kontes) and RNA was extracted into two size-fractions using the mirVana kit (Ambion). The high molecular weight fraction was further used for mRNA-seq and RNA quality was analysed using an Agilent 2100 Bioanalyzer (Agilent Technologies). First and 2nd strand cDNA synthesis, fragmentation, adapter ligation and amplification were performed using the mRNA-Seq 8-Sample Prep Kit (Illumina) according to manufacturer's instructions.

The embryos reared in 2010 were used for real-time PCR analysis. For RNA extraction from heads, embryos were dechorionated and then decapitated in front of the pectoral fin (Figure 1 B–C). Whole embryos or heads were placed in TRI Reagent (Sigma) and homogenized as described above. RNA was prepared according to manufacturer's instructions and dissolved in 30 µl RNase-free water. To minimise DNA contamination, RNA was treated with DNase (New England Biolabs). Quantity and quality of the resulting RNA were assessed using a NanoDrop ND-1000 UV/Vis-Spectrophotometer (NanoDrop Technologies). The quality of the RNA from half of the samples was further evaluated by agarose gel electrophoresis or using Bioanalyzer. All samples displayed intact 28 S and 18 S rRNA without high molecular weight genomic DNA contamination. cDNA was prepared from 200 ng of RNA using the High capacity cDNA RT kit (Applied Biosystems), according to manufacturer's protocol. The absence of genomic DNA was confirmed by preparing several samples without addition of reverse transcriptase. cDNA was diluted 5 fold in water for further use in quantitative real-time PCR.

### RNA sequencing and assembly of Arctic charr reference gene homologues

The whole mRNA transcriptome from the two 2009 charr groups (AC and SB- charr) at 4 developmental time points was sequenced, yielding single end reads of 36 base pairs. Sequencing was performed at DeCode genetics (Reykjavík, Iceland) using SOLEXA GAII technology (Illumina Inc.) and the sequencing depth ranged from 49 to 58 million reads with a mean depth of 55 Million reads per sample. The reads were pre-assembled into contigs using Velvet assembler [34], and further assembly steps were performed in CLC Genomics Workbench (CLC bio, Aarhus, Denmark). In order to obtain sequences for Arctic charr reference genes we selected likely candidates from related species, i.e. ESTs and FLIcs (full-length sequenced inserts from cDNAs) from Atlantic salmon (*Salmo salar*) and rainbow trout, (*Oncorhynchus mykiss*) [35]–[37]. The selected reference candidates were used for reference assembly of the charr homologues. The individual nucleotide mismatch score and the total mismatch score limit was set to 98% identity. All 12 consensus sequences of the charr candidate reference genes (Table 1) and the three developmental genes examined in this study have been deposited at NCBI. GenBank Accession numbers for sparc, sox9a and mmp are KC538874, JQ624876 and KC538875, respectively.

**Table 1:** The reference genes selected in this study, their abbreviation and putative function.

Gene Name	Symbol	Function	Accession no. Arctic charr
Actin, cytoplasmic 1(beta-actin)	ACTB	Cytoskeletal structure protein	JR540730
beta-2-microglobulin	b2m/B2MG	Beta chain of major histocompatibility complex	JR540731
elongation factor 1 alpha	EF1α	Protein synthesis	JR540732
Glyceraldehyde-3-phosphate dehydrogenase	G3P/GAPDH	Glycolytic protein	JR540733
Hypoxanthine-guanine phosphoribosyltransferase	HPRT	Enzyme in purine metabolic pathway	JR540734
Eukaryotic initiation factor 5A isoform 1	IF5A1	Protein synthesis	JR540735
60S ribosomal protein L7	RL7	Member of ribosome proteins	JR540736
40S ribosomal protein S9	RS9	Member of ribosome proteins	JR540737
Ribosomal Protein S20	RS20	Member of ribosome proteins	JR540738
Tubulin alpha chain	TBA	Cytoskeletal protein	JR540739
Ubiquitin-conjugating enzyme E2 L3	UB2L3	Protein degradation	JR540740
Ubiquitin	UBIQ	Protein degradation	JR540741

To quantify the expression levels of the candidate reference genes, reads were aligned to salmon mRNA sequences (total of 16727 sequences) from the NCBI-nucleotide database, using bowtie, version 0.12.7 [38]. The number of reads for each sequence was extracted using a python script. Subsequently a filter step was performed to exclude sequences that had less than 20 reads aligned and sequences to which only reads from post hatching samples aligned, in order to be able to estimate parameters in subsequent regression analysis. 15396 sequences passed this filtering step. Reads per million aligned per kilobase (RPMK-values) were calculated as expression measurements for the genes. Mean, standard deviation and coefficient of variation were calculated for the RPMK-values of the candidate reference genes

### Primer design

The assembled Arctic charr consensus sequences were used to design primers for the candidate reference and developmental genes. We aimed to make qPCR primers overlapping exon boundaries or located in separate exons (Table S1 in File S1). As the charr genome has not been sequenced but gene exon/intron boundaries are for the most part well conserved between orthologues [39], the exon/intron borders were assumed to be similar to zebrafish. The NCBI Spidey software (www.ncbi.nlm.nih.gov/spidey) was used and the consensus sequences of Arctic charr candidate genes were aligned against zebrafish homologue genes which were retrieved from the Ensemble database (http://www.ensembl.org/Danio_rerio). Primers were designed using OligoPerfect Designer (Invitrogen) and Primer Express 3.0 software (Applied Biosystems, Foster City, CA, USA). Primers were also checked for self-annealing, hetero-dimers and hairpin structures by OligoAnalyzer 3.1 (Integrated DNA Technology).

### Real-time quantitative PCR

Real-time PCR was performed in 96 well-PCR plates on an ABI 7500 real-time PCR System (Applied Biosystems) using Power SYBR green PCR Master Mix as recommended by the manufacturer (Applied Biosystems) with the exception of using 10 µl final reaction volume. Reactions were run in duplicate together with no-template control (NTC) in each run for each gene. Experimental set-up per run followed the preferred sample maximization method described by Hellemans et al. [40], in order to decrease run-to-run variation. The PCR was started with a 2 min hold at 50°C followed by a 10 min hot start at 95°C. Subsequently the amplification was performed with 40 cycles of 15 sec denaturation at 95°C and 1 min annealing/extension at 60°C. For each sample a dissociation step (60°C–95°C) was performed at the end of the amplification phase to identify a single, specific melting temperature for each primer set (Table S1 in File S1). Primer efficiencies (E) were calculated with 7 points of 2–4 fold serial dilutions using pooled cDNA (700 ng RNA input) from different developmental stages as well as different charr groups. The slope of the standard curve in the equation; E% = (10^1/slope - 1^)×100, was used for PCR efficiency calculation [41]. The range of PCR efficiencies and linear correlation coefficient (R^2^) are shown in Table S1 in File S1. The background-corrected fluorescence values from the real-time PCR were imported into LinReg PCR software [42] and the individual PCR efficiency of each reaction was determined.

### Data analysis

#### (i) Ranking candidate reference genes

First we employed three ranking analyses to detect the most stably expressed candidates. Two Excel-based programs BestKeeper [43] and NormFinder [44], as well as an improved version of the classical geNorm [45], called GeNorm^Plus^ (Biogazelle, Ghent, Netherlands). Two sources of input were used for the analysis with BestKeeper. The raw Cq values and the logarithmic N_0_ values calculated by LinReg PCR [42], which takes the individual qPCR efficiency into account. The standard deviation (S.D.) based on Cq values of the developmental stages and groups was calculated by BestKeeper to determine the expression variation for each reference gene. A standard deviation higher than 1 leads to the rejection of the candidate as reference gene. In addition, BestKeeper determines the stability of reference genes based on correlation to other candidates through calculation of BestKeeper index (B.I.). In order to decrease the effect of co-regulation in BestKeeper, we used the average B.I. for all genes compared to the first four, three and two genes with lowest standard deviations. For GeNorm and NormFinder the relative expression ratios are used as input. GeNorm measures the expression stability (*M* value) which is the mean pairwise variation between each gene and other candidates and it excludes the gene with the highest *M* value (least stable) from subsequent analysis in a stepwise manner. GeNorm^Plus^ on the other hand is able to determine the best among the last two remaining genes. GeNorm assumes candidates with *M*>1.5 as unreliable and *M*<0.5 is characteristic for very stable reference genes. GeNorm^Plus^ can also determine the optimal number of reference genes to use. For this a pairwise variation coefficient Vn/n+1 between two sequential normalisation factors (NFn and NFn+1) is calculated and extra reference genes are added until the variation drops below the recommended threshold of 0.15 [45]. NormFinder ranks the most stable genes (lowest expression stability values) based on analysis of the sample subgroups (time and charr group) and estimation of inter- and intra-group variation in expression levels.

Secondly an analysis of variance (ANOVA) followed by post hoc Tukey's honest significant difference (HSD) test was implemented using relative expression ratios in R [46] (http://www.r-project.org/) with developmental time points and progeny groups as categorical variables. Relative expression ratios were calculated using primer efficiencies (E). For this the highest expressed sample point (the lowest Cq = *Min Cq*) in each primer pair was set to one and the other sampling points were calculated in relation to Min Cq, according to E ^ΔCq^, where ΔCq = Min Cq–Cq sample. The best reference genes were considered those that showed no (or very little) significant difference in relative expression among time points or embryo groups.

#### (ii) Comparison of qPCR and RNA-seq data

To compare the RNA-seq and qPCR expression data we tested the correlation between RPMK (transformed to a log2-scale) and quantification cycle (Cq)-values, for the 8 corresponding samples for group and relative age (Figure 1A, lower panel), using linear regression on the RPMK values.

A generalized linear model was applied to the raw RNA-seq read counts and tested for group and time effect with a likelihood ratio test using the edgeR-package in R [47]. The coefficient of variation was also calculated as a stability measure.

#### (iii) Consistency of normalisation

Expression levels of the three putative developmental genes, sparc, mmp2, and sox9a, in four charr groups at three developmental time points were calculated using either individual reference genes or a combination of reference genes (i.e. geometric average Cq of two and three reference genes) for normalisation. Fold changes were calculated by comparing expression in three charr morphs from Thingvallavatn (SB, LB and PL) to expression in aquaculture charr. The consistency of normalisation with the three reference genes was examined running three full model ANOVAs (one for each developmental gene), especially examining the interaction terms for (morph)×(developmental time point)×(reference gene) and (morph)×(reference gene). Furthermore we tested for consistency in reflecting variation (as measured by coefficients of variation, CVs, for two biological replicates) of the mean expression levels for each developmental gene, morph and developmental time point. This was done by testing for positive correlations of the CVs between the three reference genes. Statistical differences between benthic (SB and LB) and pelagic (PL and AC) groups in the expression of target genes were determined using Student's t test.

## Results

In order to identify key regulator elements responsible for the phenotypic differences in the charr morphotypes we sequenced the transcriptome of two charr groups at four points during development. We focused on the developmental stages covering cartilage formation and the beginning of ossification (Figure 1). Transcriptome sequencing was carried out on RNA from whole embryos. In the present study this data was used to examine the expression of selected reference genes and to compare these results to the qPCR data.

Twelve possible reference genes originating from independent pathways were selected for validation, based on previous studies in other fish species [23]–[29] (Table 1). The sequences of these genes from salmon or other fish species were used for reference based assembly of the corresponding charr mRNA sequences. Quantitative real-time PCR primers were designed (Table S1 in File S1) and, with the exception of the GAPDH primers, all primer efficiencies were shown to lie within the 90–110% range. Melting curve analysis revealed the absence of primer dimers and different size of amplification products for all primer pairs

### Transcription profiling of candidate reference genes

**Figure 2:**
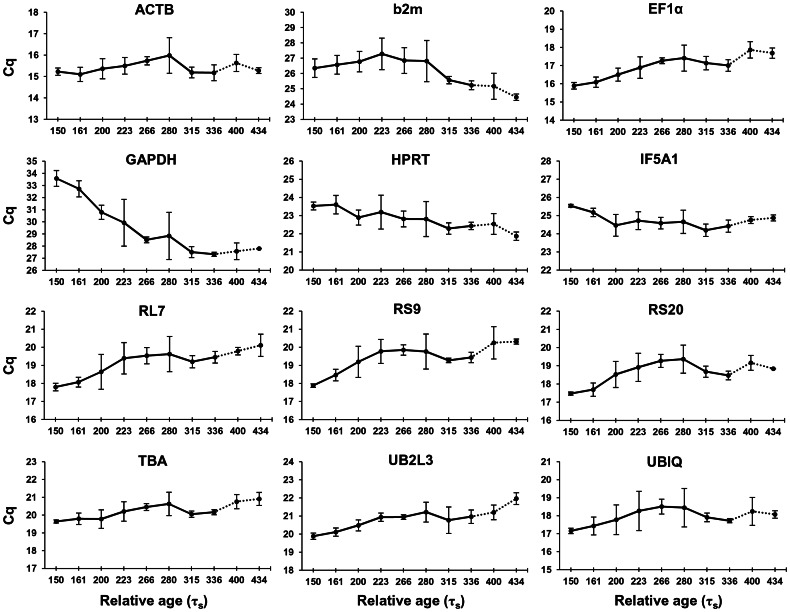
Expression levels of reference genes in the head of four charr groups during development. Expression profiles of 12 candidate reference genes based on quantitative real time PCR performed on embryonic heads from four charr groups at the relative ages 150 to 434 τ_s_. Expression levels are shown as mean Cq (quantification cycle) values in the four charr groups at corresponding relative age, except for the two last time points (dashed line), which are based on samples of only two groups(AC and PL charr). Error bars represent standard deviation.

Quantitative real-time PCR for the 12 reference gene candidates was performed on cDNA generated from head and whole embryo samples as described in Figure 1. Candidate reference gene expression levels during head development were profiled in the charr groups using Cq values (Figure 2). Out of the 12 genes, GAPDH showed increasing expression during development, which, combined with high primer efficiency, led us to reject this gene as a reference gene.

The remaining eleven candidates covered a broad range of expression levels, varying from ACTB, with the highest expression (lowest Cq) (Figure S1A in File S2,), to b2m with the lowest expression (highest Cq). When the raw Cq values were transformed to relative expression ratios, seven genes showed significant difference (*P*<0.05) in expression between head and whole embryo (Figure S1B in File S2), illustrating the differences in expression of genes between different body parts and the importance of validating reference genes in the tissue of interest. Interestingly some genes had lower, other higher expression in head compared to whole embryo indicating the robustness of the reference genes chosen.

### Reference gene analyses and ranking

The candidate reference genes were ranked using three known algorithms (BestKeeper, GeNorm and NormFinder) and based on standard deviation (SD). For simplicity the ranking of genes using all 4 methods is only shown in Table 2, while detailed results can be found in supplementary tables (Tables S2 in File S1). In charr heads TBA was found to be the most stably expressed reference gene across all charr groups as well as within each group (Table S3 in File S1). ACTB was shown to be the second most stable gene in both analyses and UBIQ and UB2L3 were among the 4 best reference genes both in heads in general and when examining each group separately. GeNorm suggested the use of only two reference genes to be sufficient for accurate normalisation (Figure S2 in File S2). This data reflects the high stability of the candidate reference genes expression and suggests that TBA and ACTB are sufficient and suitable reference genes to quantify gene expression in Arctic charr heads.

**Table 2:** Ranking of the candidate reference genes in Arctic charr head homogenates using BestKeeper (BK), geNorm (gN), NormFinder (Nf) and standard deviation (SD).

Gene	BK	gN	Nf	SD
ACTB	3	8	1	2
b2m	9	11	11	11
EF1α	5	3	7	5
HPRT	10	10	4	6
IF5A1	11	9	5	3
RL7	7	5	10	10
RS9	4	4	9	9
RS20	6	6	8	8
TBA	1	1	2	1
UB2L3	8	2	6	4
UBIQ	2	7	3	7

**Figure 3:**
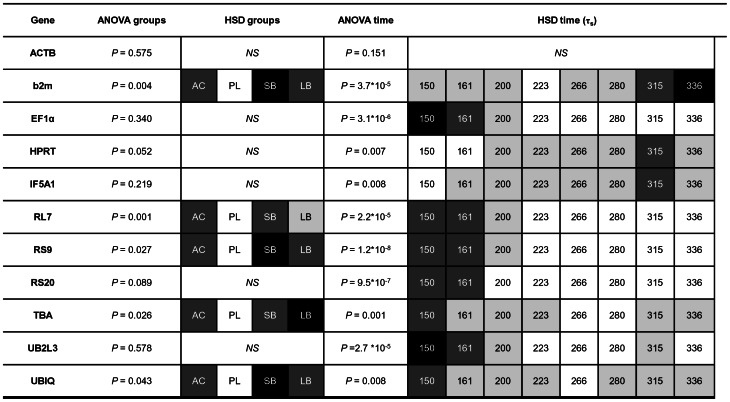
Candidate reference gene expression differences and patterns in the head of Arctic charr groups during development. Relative expression ratios, calculated from the qPCR data, were subjected to an analysis of variance (ANOVA) to test the expression differences amongst four charr groups and eight time points (numbers are relative age in τ_s_). Subsequently a post hoc Tukey's honestly significant difference test (HSD) was performed to analyse the expression pattern of candidates in groups and during development. White boxes represent low expression, while black boxes represent high expression. A two or more shade difference in the boxes represents significant different expression between the samples (alpha = 0.05). *NS* = not significant.

We further performed a two-way ANOVA followed by a Tukey's test (Figure 3) to select reference genes which are not expressed at significantly different levels between group/time – the main criteria for a stable reference gene. These analyses identified six candidate reference genes that are stably expressed between groups, but of those only ACTB showed constant expression during the developmental stages examined. The post hoc Tukey's test revealed the expression pattern of the genes over the time examined. Several genes, e.g., ribosomal protein genes, EF1α and UB2L3, were found to be highly expressed at the earlier stages, while others were more highly expressed later in development e.g. b2m. Based on these results ACTB was found to be the overall most stable reference gene both over time and between the 4 different groups.

### Testing consensus of transcriptome and qPCR data

**Figure 4:**
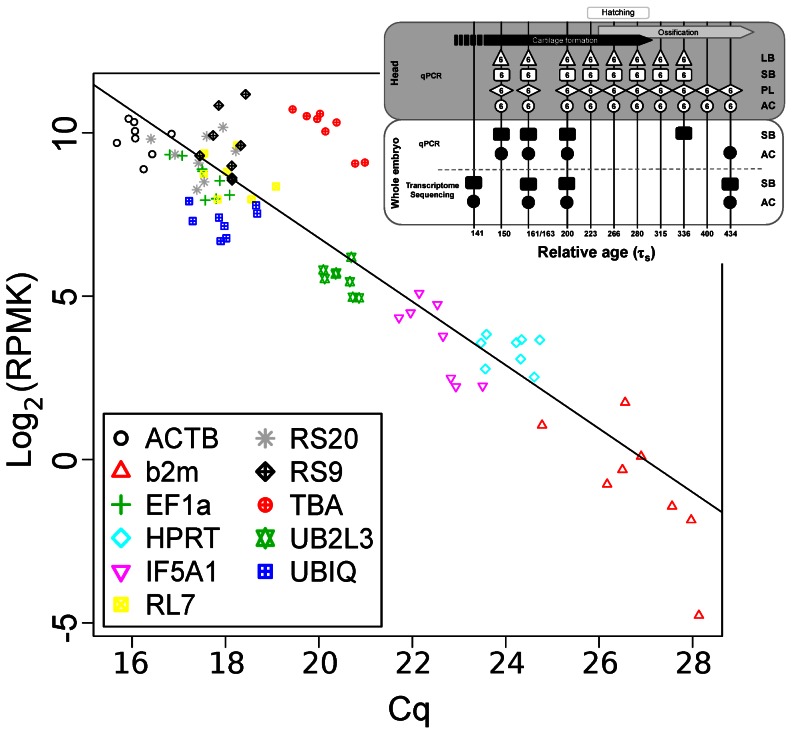
Comparison between expression values from RNA-seq and qPCR. Reads per million aligned per kilobase (RPMK) transformed to a log2-scale were plotted against equivalent Cq-values for eleven candidate reference genes. The compared samples were from the same groups and at the same or similar relative age (insert, black spots represent samples used for analysis). The line is a least squares linear fit to the data (y = 26.23–0.97x, R^2^ = 0.815).

RNA-seq and qPCR were used to estimate the expression levels of the candidate reference genes in whole embryos (samples used see Figure 4 insert) and the two methods were compared. As expected the expression estimates from RNA-seq data correlated significantly with the expression estimates from qPCR (p<1^−10^) (Figure 4).

Candidate reference genes were ranked for expression stability in whole embryos using both the qPCR and the RNA-seq data (Table 3). Overall UB2L3 and ACTB were found to be most stable. Furthermore, UB2L3 showed no significant differences between groups or during development, as determined using a likelihood-ratio test, qualifying this gene as the best reference gene in whole embryos. Interestingly UB2L3, which was one of the four best reference genes in head samples (Table 2), is also the best reference gene for comparing head and whole embryo gene expression (Figure S1B in File S2).

**Table 3:** Ranking of reference gene candidates, based on stability of expression in Arctic charr whole embryos using either qPCR or RNA-seq.

	qPCR		RNA-seq		
Gene	Nf	SD	C.V.	p M	p T
ACTB	2	3	4	*	-
b2m	11	11	11	**	**
EF1α	3	4	6	-	-
HPRT	5	5	3	-	*
IF5A1	10	10	9	-	**
RL7	9	9	7	-	-
RS9	1	2	8	-	*
RS20	7	8	10	*	**
TBA	8	6	5	-	-
UB2L3	4	1	1	-	-
UBIQ	6	7	2	-	-

Abbreviations: Nf _ NormFinder, SD = standard deviation, C.V. = coefficient of variation (used to rank), p M = significant differences between morphological groups, p T = significant differences between developmental time points.

**p = <0.01; * = p<0.05; - = no significant difference.

### Consistency of normalised expression levels of three craniofacial target genes

**Figure 5:**
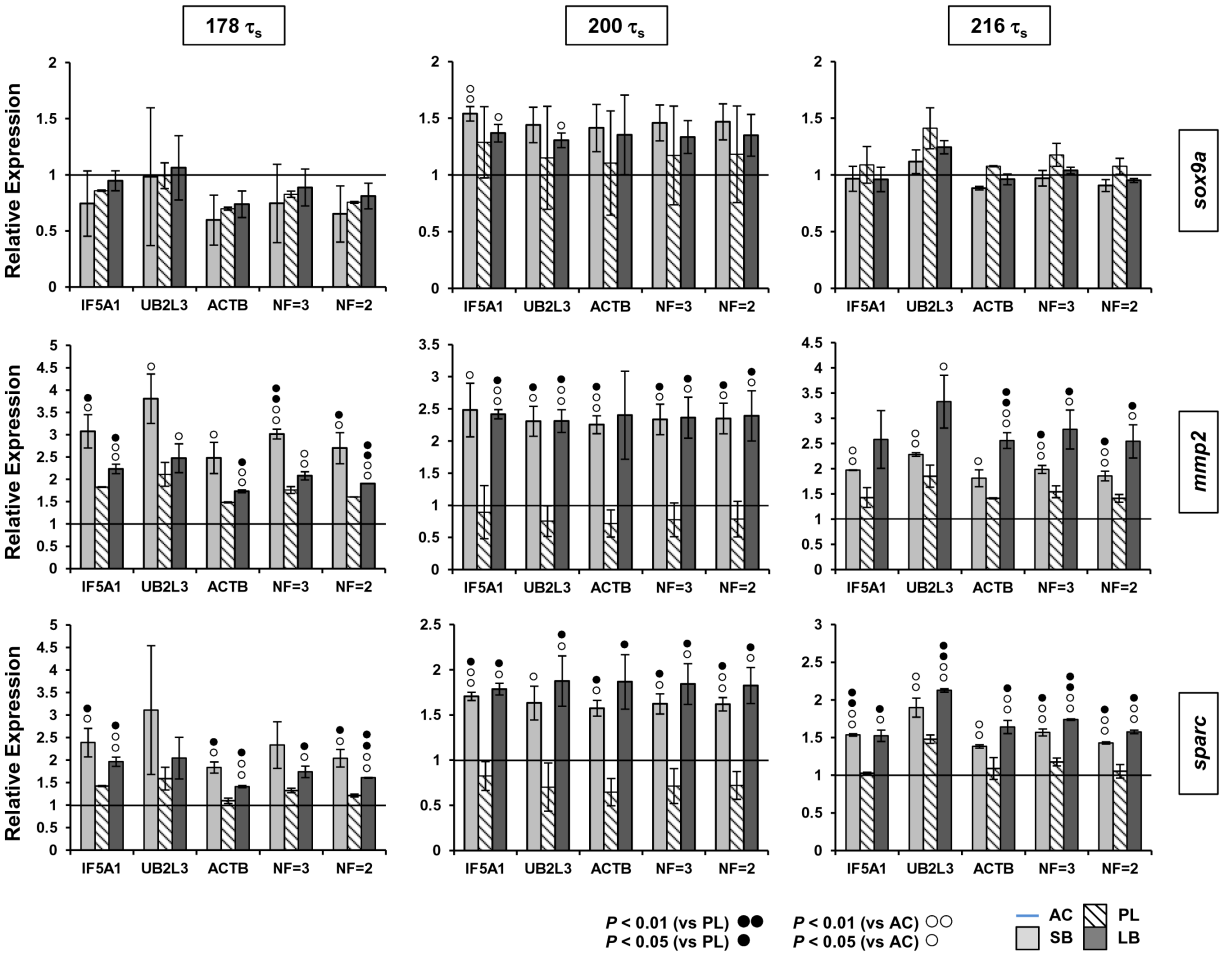
Comparison of different reference genes for normalising the expression of sox9a, mmp2 and sparc in charr heads at three embryonic stages. Expression of sox9a, mmp2 and sparc was examined with qPCR and normalised using either individual or two combinations of reference genes. Normalisation factors (NF) were based on geometric means of either two or three genes (NF = 2: ACTB and IF5A1; NF = 3: ACTB, IF5A1 and UB2L3). In each analysis (panel column) relative expression levels of the three genes in small benthivorous (SB), planktivorous (PL) and large benthivorous charr (LB), are compared to expression levels in aquaculture (AC) charr (horizontal line) at the same embryonic stage. Statistical differences of SB or LB gene expression versus expression in either PL (black circles) or AC (white circles) are indicated. Error bars represent standard deviation calculated from two biological replicates. Each biological replicate contains homogenate of six heads.

For a test-run of our validated qPCR reference genes, we selected three genes which have a well established craniofacial expression pattern during zebrafish development [48]–[50] and showed expression differences between charr groups in our transcriptome data. Arctic charr homologues of sparc, mmp2 and sox9a all showed elevated expression in SB compared to AC at 200 τ_s_ (unpublished data). The expression of these genes was examined in the head of all four charr groups and at three developmental time points. To normalise the qPCR data, we used ACTB, UB2L3 and IF5A1 separately, the geometric average expression of all three genes (NF = 3), or the geometric average expression of IF5A1 and ACTB alone (NF = 2) (Figure 5). The three ANOVAs of normalised expression values show that expression patterns among developmental time points and morphs are the same for all three reference genes. P values for the three-way interaction term involving the reference genes were non-significant in all three ANOVAs (P = 0.9961, P = 0.5895, P = 0.7715 for sox9a, mmp2 and sparc, respectively). Furthermore, the CVs of mean expression values showed significant correlations among reference genes (Correlation coefficients and Bonferroni adjusted P values: r = 0.692, P<0.001 for IF5A1 versus UB2L3; r = 0.556, P<0.008 for IF5A1 versus ACTB; r = 0.557, P<0.008 for ACTB versus UB2L3). Interestingly, sparc and mmp2 showed significantly higher expression in the heads of the two benthic morphs (SB and LB) compared to the AC and PL groups at all three time points (Figure 5). Standard deviations of the normalised expression levels were generally low and the expression differences between morphotypes were consistent among the three reference genes (Figure 5) showing the robustness of their use for normalisation. In the case of sparc all normalisation methods detected a 1.7 fold difference in expression levels between the morphotypes. Hence, we can conclude that all three genes are suitable as reference genes for qPCR studies of Arctic charr development. Although the use of one reference gene already gave consistent results, the geometric mean of two or three reference genes further decreased variations in the relative expression.

## Discussion

Numerous studies have discussed the importance of proving the stability of reference genes under the relevant study conditions [30], [32]. Previously reference genes validated in salmon and trout have been used to study charr gene expression [51], [52]. We examined several of these genes, but excluded them in further analyses due to technical problems, such as low primer efficiencies and non-specific amplification (data not shown). One of our aims in this study was to select genes from a wide variety of pathways in order to ensure a robust normalisation strategy for further gene expression analysis. This will enable us to detect small changes in the expression of developmental genes important for the formation of the craniofacial morphology of Arctic charr. Using a combination data from RNA sequencing and qPCR we have established a suite of reference genes suitable for studying gene expression in charr embryos. All of the candidate reference genes except GAPDH can be considered suitable for comparative analyses of qPCR data. Overall ACTB, TBA, UBIQ and UB2L3 were found to be the most stably expressed genes, but the ranking of the different genes varied according to body part and charr group examined, as well as by the method of analysis used. Other studies of reference genes have discussed the tendency of reference gene validation programs to rank co-regulated genes with similar expression patterns as the most stably expressed genes [23], [44]. In this study we found that GeNorm and BestKeeper rankings favour genes that are co-regulated, whereas genes such as b2m, HPRT and IF5A1 are often at the bottom of the rankings. This is due to the fact that these three genes have expression patterns that are entirely different from the rest of the candidates (Figure 3) affecting the ranking with these two algorithms. Our aim was to select reference genes which are not co-regulated. EF1α and the ribosomal protein genes, for example, have a role in protein biosynthesis and show very similar expression patterns. Similarly the two genes involved in protein degradation, *i.e.* UBIQ and UB2L3, conform to the expression of ribosomal protein genes. Genes like b2m, HRPT and IF5A1 on the other hand were found to be more highly expressed at the later points of development examined here. Therefore either HPRT or IF5A1 in combination with ACTB, TBA or UB2L3 would be good reference genes. This is confirmed in our pilot study of the expression profile of the three developmental genes. Our approach using ANOVA and post hoc HSD tests to analyse expression profiles of genes provides important advances over the commonly used programs for reference gene selection and validation. This is illustrated when considering TBA as reference gene. TBA was found to be the highest ranking gene in charr heads, but showed significantly different expression among groups and the relative ages examined (Figure 3). This suggests that although TBA is very stable within each charr group, it might not be the most suitable reference gene when comparing gene expression between charr morphs/groups. For this reason we did not include TBA in our pilot analysis of developmental genes. These findings illustrate the importance of understanding the background of the algorithms used, in order to choose reference genes and to clarify which genes are suitable for the task at hand, instead of relying on one method of reference gene selection.

When comparing the transcriptome and qPCR data we found that in general the two methods recorded similar gene expression levels. An exception to this is TBA with higher transcript levels seen in the RNA-seq data, than determined by qPCR (Figure 4). This result might be explained by the presence of gene paralogues. Salmonids, including Arctic charr, have undergone a recent genome duplication event [53] and this has led to the evolution of gene paralogues [54], [55]. The TBA primers used here, only bind one of at least 3 paralogues of TBA in charr and this may have led to an underestimation of the expression of TBA using qPCR compared to the sequenced reads (Figure 4).

When examining our sequencing data in detail we found that all reference genes except RS20 and HRPT have paralogues in Arctic charr (unpublished data). In contrast to TBA these other primer pairs are thought to amplify all paralogues for the respective gene. The amplification of several paralogues with a single primer pair could explain the high expression stability observed for most genes and interestingly this did not result in a broader melting curve for the PCR products, reflecting identical lengths and GC content of the paralogues. These results underline the importance of considering the presence of paralogues when studying gene expression in salmonids, but for selection of stable qPCR references their presence may actually be an advantage.

Further evaluations of the consistency of normalisation using three of our newly validated reference genes (IF5A1, UB2L3 and ACTB) were made in a pilot study examining the expression of three developmental genes (sox9a, sparc and mmp2) at three time points in developing Arctic charr heads. The analyses showed that each of the three reference genes could be used individually with consistent results, but the use of two or three reference genes decreased the small observed variation in expression even further. Therefore, in future comparative studies of the development of divergent trophic morphologies in Arctic charr, we will use the geometric mean of ACTB and IF5A1.

The developmental results of the pilot study are of considerable interest. While sox9a expression varied significantly through time, variation among morphs was not significant. Sox9 is a member of the Sry-related HMG-box gene family and encodes a transcription factor with an important and highly conserved role in cartilage formation [56]–[60], Two co-orthologues of sox9 (sox9a/b) with overlapping expression pattern have been reported during craniofacial cartilage formation of teleost fish [50], [61]–[63]. Sox9a was differentially expressed in our transcriptome analysis, but we could not detect similar differences with qPCR analysis. This might be caused by the fact that transcriptome sequencing was performed on whole embryos, whereas qPCR was focused on charr heads and sox9a might not be differentially expressed in the head.

Expression of sparc and mmp2 varied significantly both in time and among the morphs. Sparc/osteonectin is a highly conserved collagen-binding glycoprotein which plays important roles in extracellular matrix (ECM) remodelling and craniofacial morphogenesis [49], [64]–[66]. Similarly, matrix metalloproteases, including mmp2, have important roles during craniofacial morphogenesis through precise regulation of ECM degradation [48], [67]. Sparc has been suggested to act downstream of sox9 during cartilage formation of the pharyngeal arches [49]. In our data, however, sparc expression levels, which are higher in the benthic morphs, do not go hand in hand with sox9a levels (Figure 5). An association between sparc up-regulation and increased mmp2 expression and activity, has been shown in various studies [64], [68]–[73]. In the present study both genes are consistently expressed at higher levels in the head of benthic than pelagic groups, suggesting a role of these genes in the observed differences in trophic morphology between the charr morphotypes.

In conclusion we have, using data from transcriptome sequencing and qPCR, identified several suitable reference genes for the analysis of gene expression in developing Arctic charr embryos. Furthermore, we have used these to confirm putative expression differences between the charr morphotypes in two craniofacially expressed genes. The tools generated here will be of great use in further analyses of gene expression in Arctic charr embryogenesis and will be instrumental in our search for genes that play key roles in inducing different trophic morphotypes. Finally, the use of ANOVA for reference gene selection as we have demonstrated will be useful for validation of reference genes in other species.

## Supporting Information

File S1 **Contains: Table S1 qPCR Primer sequences and information. Table S2 A–F Descriptive statistical analysis of the candidate reference gene expression in Arctic charr using three algorithms. Table S3 Ranking of the candidate reference genes in the heads or the different Arctic charr groups using NormFinder (Nf) and Standard deviation (SD).**
(DOC)

File S2 **Contains: Figure S1 Comparison of expression levels of the eleven candidate reference genes in heads and whole embryos using qPCR.** The genes are ranked from left to right as most to least differentially expressed between whole embryos (whole) and head (corresponding P-values are shown below the x-axis). Insert in A displays samples (black filled spots) used in both analyses. (**A**) Boxplot shows the range of Cq values for each candidate reference gene in whole embryo (white) and head (gray) homogenates. Displayed are the median, the 25th and 75th percentiles and the minimum and maximum Cq values for each gene. (**B**) Relative quantity for each reference gene candidate in whole embryos (open circles) and head (grey diamond) homogenates. The whiskers represent ±0.95 confidence interval of the mean. **Figure S2 Optimal number of reference genes for normalisation.** Genorm^PLUS^ was used to determine the optimal number of reference genes in head and whole embryo homogenates. LB: large benthic; SB: small benthic/dwarf; PL: planktivorous; AC: aquaculture. Average pair-wise variations (V_n/n+1_) were calculated using the genes ranked according to GeNorm. The recommended cut-off value of 0.15 is shown by a dashed line and below this line the benefit of using an extra reference gene is limited.
(DOCX)
