# Supplementary material for: Validation of Reference Genes for Expression Studies during Craniofacial Development in Arctic Charr
Source: PLoS One. 2013 Jun 13;8(6):e66389. doi: 10.1371/journal.pone.0066389 (PMC3681766; doi:10.1371/journal.pone.0066389)
Supplement: File S1 — Contains: Table S1 qPCR Primer sequences and information. Table S2 A–F Descriptive statistical analysis of the candidate reference gene expression in Arctic charr using three algorithms. Table S3 Ranking of the candidate reference genes in the heads or the different Arctic charr groups using NormFinder (Nf) and Standard deviation (SD). (DOC) [file pone.0066389.s001.doc]

**File S1:**

**Table S1. qPCR P**rimer sequences and information

| **Gene** | **Primer Sequence (5’- 3’)** | **Product Size (bp)** | **Efficiency (E %)** | **R^2^ *** | **Melting Temperature (°C) Head** | **Melting Temperature (°C) Whole Embryo** | **Location** (Exon No.)** |
| --- | --- | --- | --- | --- | --- | --- | --- |
| **ACTB** | F GAAGATCAAGATCATCGCCC | 122 | 95.5 | 0.998 | 80.5 ± 0.7 | 80.52 ± 0.7 | 4:5 |
|  | R CAGACTCGTCGTACTCCTGCT |  |  |  |  |  | 5 |
| **b2m** | F CGAACAGGGATGGCAGTT | 105 | 95.5 | 0.998 | 80.92 ± 0.4 | 81.31 ± 0.2 | 1 |
|  | R TAGGTCTTCAGATTCTTCAGGTGG |  |  |  |  |  | 1 |
| **EF1α** | F GAAGATCGGCTATAACCCTGC | 111 | 94.6 | 0.998 | 81.36 ± 0.4 | 81.11 ± 0.1 | 3 |
|  | R ACCTTCCATCCCTTGAACC |  |  |  |  |  | 4 |
| **GAPDH** | F GGTCTGATGAGCACCGTTC | 110 | 114.8 | 0.96 | 81.3 ± 0.3 | 81.46 ± 0.2 | 5:6 |
|  | R GCAGGGATGATGTTCTGGC |  |  |  |  |  | 6 |
| **HPRT** | F TTCTCAAACAGTACAACCCAAAAA | 95 | 96.3 | 0.994 | 76.57 ± 0.4 | 76.05 ± 0.2 | 5 |
|  | R TCCTATGAAGTCTGGTGTGTAGC |  |  |  |  |  | 6:7 |
| **IF5A1** | F GGCTTCGTGGTGCTGAAG | 91 | 91.5 | 0.999 | 80.76 ± 0.6 | 81.1 ± 0.1 | 1 |
|  | R CCATGTGGACCTTAGCGTG |  |  |  |  |  | 1:2 |
| **RL7** | F CATCAGGATCAGGGGTATCAA | 109 | 91.9 | 0.998 | 81.29 ± 0.2 | 80.67 ± 0.5 | 1:2 |
|  | R AGCCTTGTTCAGTTTGACGAA |  |  |  |  |  | 2 |
| **RS9** | F GAGGTGTGGAGGGTGAAGTT | 113 | 95.4 | 0.998 | 82.03 ± 0.4 | 82.18 ± 0.4 | 2 |
|  | R CTGAGCAGGGCGTTACCTT |  |  |  |  |  | 2:3 |
| **RS20** | F AGCCGCAACGTCAAGTCT | 110 | 96.7 | 0.999 | 79.9 ± 0.8 | 80.5 ± 0.3 | 1 |
|  | R CGCAGAGTCTTTGTGGGC |  |  |  |  |  | 2:3 |
| **TBA** | F GTCACTACACCATTGGCAAAGA | 104 | 103.5 | 0.994 | 78.9 ± 0.4 | 79.02 ± 0.2 | 2 |
|  | R GCTGTGGAAGATGAGGAATCC |  |  |  |  |  | 3 |
| **UB2L3** | F CGAGAAGGGACAGGTGTGTC | 96 | 93.4 | 0.999 | 79.62 ± 0.3 | 79.5 ± 0.3 | 3 |
|  | R ACCAACGCAATCAGGGACT |  |  |  |  |  | 4 |
| **UBIQ** | F GACTACAACATCCAGAAAGAGTCCA | 120 | 92.6 | 0.999 | 79.4 ± 0.3 | 79.36 ± 0.1 | 2:3 |
|  | R GCGGCAGATCATTTTGTC |  |  |  |  |  | 3 |
| **sox9a** | F CCTGAGTGGAGGTGGAGG | 74 | 102.4 | 0.999 | 79.3 ± 0.3 | - | N-S |
|  | R GCTCCGTCTTGATCTGAGTG |  |  |  |  |  |  |
| **sparc** | F GTTCTGGTCACCCTGTACGAG | 100 | 91.6 | 0.999 | 79.9 ± 0.1 | - | N-S |
|  | R GCAGTCTCTTCTCATTCTCATAGATC |  |  |  |  |  |  |
| **mmp2** | F ATGGATGGAGAGGCTGACATC | 110 | 93.7 | 0.999 | 79.73 ± 0.3 | - | N-S |
|  | R GGTCCAGGAGCAAAGGCAT |  |  |  |  |  |  |

(*)R^2^, correlation coefficient of the slope of the standard curve (**) Putative exon borders based on zebrafish homologues. The numbers are referring to the exon(s) in which the primers are located, (n:n) and (N-S) indicate “exon/exon” boundaries and “non-specified”, respectively.

**Table S2A. Descriptive statistical analysis of the candidate reference gene expression in Arctic charr heads using** three algorithms.

| **Algorithm** |  | **ACTB** | **b2m** | **EF1a** | **GAPDH** | **HPRT** | **IF5A1** | **RL7** | **RS9** | **RS20** | **TBA** | **UB2L3** | **UBIQ** |
| --- | --- | --- | --- | --- | --- | --- | --- | --- | --- | --- | --- | --- | --- |
| BestKeeper | B.I. all (r) | 0.795 | 0.587 | 0.708 | -0.039 | 0.447 | 0.188 | 0.735 | 0.773 | 0.77 | 0.823 | 0.613 | 0.932 |
|  | B.I. 4 genes (r) | 0.794 | 0.278 | 0.784 | -0.235 | 0.195 | 0.167 | 0.785 | 0.782 | 0.751 | 0.932 | 0.801 | 0.815 |
|  | B.I. 3 genes (r) | 0.838 | 0.441 | 0.646 | 0.017 | 0.42 | 0.383 | 0.618 | 0.614 | 0.644 | 0.822 | 0.557 | 0.778 |
|  | B.I. 2 genes (r) | 0.917 | 0.392 | 0.787 | -0.254 | 0.2 | -0.01 | 0.747 | 0.763 | 0.76 | 0.885 | 0.716 | 0.869 |
|  | overall B. I.* (r) | 0.836 | 0.425 | 0.731 | -0.128 | 0.316 | 0.182 | 0.721 | 0.733 | 0.731 | 0.866 | 0.672 | 0.849 |
|  | S.D. | 0.476 | 0.951 | 0.643 | 2.411 | 0.674 | 0.554 | 0.872 | 0.822 | 0.789 | 0.472 | 0.558 | 0.742 |
| GeNorm | M | 0.494 | 0.742 | 0.378 | 1.042 | 0.663 | 0.591 | 0.429 | 0.418 | 0.451 | 0.354 | 0.366 | 0.463 |
| NormFinder | stability | 0.13 | 0.584 | 0.398 | 1.753 | 0.359 | 0.374 | 0.516 | 0.476 | 0.403 | 0.2 | 0.39 | 0.236 |

Abbreviations: S.D. = Standard deviation, B.I. = BestKeeper Index, *r* = Pearson product-moment correlation coefficient.

*average B.I.

**Table S2B. Descriptive statistical analysis of the candidate reference gene expression in aquaculture charr heads using** three algorithms.

| **Algorithm** |  | **ACTB** | **b2m** | **EF1a** | **GAPDH** | **HPRT** | **IF5A1** | **RL7** | **RS9** | **RS20** | **TBA** | **UB2L3** | **UBIQ** |
| --- | --- | --- | --- | --- | --- | --- | --- | --- | --- | --- | --- | --- | --- |
| BestKeeper | B.I. all (r) | 0.748 | 0.738 | 0.62 | 0.166 | 0.238 | 0.201 | 0.231 | 0.356 | 0.63 | 0.608 | 0.601 | 0.762 |
|  | B.I. 4 genes (r) | 0.706 | 0.291 | 0.905 | -0.42 | -0.375 | -0.175 | 0.65 | 0.605 | 0.954 | 0.905 | 0.864 | 0.905 |
|  | B.I. 3 genes (r) | 0.384 | 0.009 | 0.985 | -0.638 | -0.539 | -0.307 | 0.882 | 0.815 | 0.935 | 0.957 | 0.947 | 0.868 |
|  | B.I. 2 genes (r) | 0.393 | 0.002 | 0.957 | -0.618 | -0.507 | -0.239 | 0.87 | 0.768 | 0.912 | 0.96 | 0.958 | 0.84 |
|  | overall B. I.* (r) | 0.558 | 0.260 | 0.867 | -0.378 | -0.296 | -0.130 | 0.658 | 0.636 | 0.858 | 0.858 | 0.843 | 0.844 |
|  | S.D. | 0.463 | 1.076 | 0.401 | 2.258 | 0.665 | 0.549 | 0.588 | 0.599 | 0.695 | 0.349 | 0.352 | 0.465 |
| GeNorm | M | 0.397 | 0.68 | 0.16 | 0.957 | 0.584 | 0.497 | 0.276 | 0.31 | 0.34 | 0.168 | 0.173 | 0.214 |
| NormFinder | stability | 0.162 | 0.592 | 0.26 | 1.602 | 0.385 | 0.333 | 0.489 | 0.462 | 0.462 | 0.222 | 0.228 | 0.242 |

Abbreviations: S.D. = Standard deviation, B.I. = BestKeeper Index, *r* = Pearson product-moment correlation coefficient.

*average B.I.

**Table S2C. Descriptive statistical analysis of the candidate reference gene expression in planktivorous charr heads using** three algorithms.

| **Algorithm** |  | **ACTB** | **b2m** | **EF1a** | **GAPDH** | **HPRT** | **IF5A1** | **RL7** | **RS9** | **RS20** | **TBA** | **UB2L3** | **UBIQ** |
| --- | --- | --- | --- | --- | --- | --- | --- | --- | --- | --- | --- | --- | --- |
| BestKeeper | B.I. all (r) | 0.86 | 0.95 | 0.813 | 0.149 | 0.736 | 0.409 | 0.838 | 0.877 | 0.926 | 0.972 | 0.866 | 0.954 |
|  | B.I. 4 genes (r) | 0.939 | 0.785 | 0.962 | -0.23 | 0.465 | 0.071 | 0.919 | 0.903 | 0.958 | 0.968 | 0.958 | 0.926 |
|  | B.I. 3 genes (r) | 0.958 | 0.832 | 0.914 | -0.131 | 0.552 | 0.168 | 0.879 | 0.875 | 0.939 | 0.982 | 0.947 | 0.915 |
|  | B.I. 2 genes (r) | 0.876 | 0.799 | 0.915 | -0.161 | 0.543 | 0.129 | 0.926 | 0.932 | 0.959 | 0.988 | 0.987 | 0.933 |
|  | overall B. I.* (r) | 0.908 | 0.842 | 0.901 | -0.093 | 0.574 | 0.194 | 0.891 | 0.897 | 0.946 | 0.978 | 0.940 | 0.932 |
|  | S.D. | 0.668 | 1.266 | 0.835 | 2.562 | 0.820 | 0.650 | 1.020 | 1.076 | 1.012 | 0.600 | 0.602 | 1.069 |
| GeNorm | M | 0.442 | 0.53 | 0.336 | 1.01 | 0.602 | 0.675 | 0.228 | 0.223 | 0.238 | 0.411 | 0.39 | 0.278 |
| NormFinder | stability | 0.25 | 0.401 | 0.403 | 1.845 | 0.295 | 0.462 | 0.472 | 0.456 | 0.357 | 0.105 | 0.27 | 0.335 |

Abbreviations: S.D. = Standard deviation, B.I. = BestKeeper Index, *r* = Pearson product-moment correlation coefficient.

*average B.I.

**Table S2D. Descriptive statistical analysis of the candidate reference gene expression in small benthic charr heads using** three algorithms.

| **Algorithm** |  | **ACTB** | **b2m** | **EF1a** | **GAPDH** | **HPRT** | **IF5A1** | **RL7** | **RS9** | **RS20** | **TBA** | **UB2L3** | **UBIQ** |
| --- | --- | --- | --- | --- | --- | --- | --- | --- | --- | --- | --- | --- | --- |
| BestKeeper | B.I. all (r) | 0.811 | -0.313 | 0.872 | -0.651 | -0.583 | -0.735 | 0.897 | 0.806 | 0.775 | 0.86 | 0.828 | 0.979 |
|  | B.I. 4 genes (r) | 0.867 | -0.003 | 0.743 | -0.593 | -0.492 | -0.81 | 0.822 | 0.796 | 0.614 | 0.831 | 0.766 | 0.923 |
|  | B.I. 3 genes (r) | 0.892 | 0.174 | 0.629 | -0.479 | -0.372 | -0.797 | 0.704 | 0.718 | 0.449 | 0.742 | 0.635 | 0.809 |
|  | B.I. 2 genes (r) | 0.896 | 0.431 | 0.339 | -0.075 | -0.03 | -0.639 | 0.324 | 0.344 | 0.079 | 0.338 | 0.266 | 0.518 |
|  | overall B. I.* (r) | 0.867 | 0.072 | 0.646 | -0.450 | -0.369 | -0.745 | 0.687 | 0.666 | 0.479 | 0.693 | 0.624 | 0.807 |
|  | S.D. | 0.430 | 0.358 | 0.653 | 2.488 | 0.471 | 0.527 | 0.742 | 0.759 | 0.707 | 0.434 | 0.729 | 0.460 |
| GeNorm | M | 0.429 | 0.512 | 0.37 | 1.018 | 0.599 | 0.669 | 0.317 | 0.351 | 0.385 | 0.292 | 0.334 | 0.293 |
| NormFinder | stability | 0.093 | 0.247 | 0.371 | 1.919 | 0.35 | 0.444 | 0.447 | 0.478 | 0.439 | 0.167 | 0.441 | 0.144 |

Abbreviations: S.D. = Standard deviation, B.I. = BestKeeper Index, *r* = Pearson product-moment correlation coefficient.

*average B.I.

**Table S2E. Descriptive statistical analysis of the candidate reference gene expression in large benthic charr heads using** three algorithms.

| **Algorithm** |  | **ACTB** | **b2m** | **EF1a** | **GAPDH** | **HPRT** | **IF5A1** | **RL7** | **RS9** | **RS20** | **TBA** | **UB2L3** | **UBIQ** |
| --- | --- | --- | --- | --- | --- | --- | --- | --- | --- | --- | --- | --- | --- |
| BestKeeper | B.I. all (r) | 0.887 | 0.493 | 0.701 | -0.456 | 0.142 | -0.434 | 0.833 | 0.791 | 0.271 | 0.797 | 0.617 | 0.893 |
|  | B.I. 4 genes (r) | 0.859 | 0.44 | 0.696 | -0.42 | 0.178 | -0.412 | 0.801 | 0.747 | 0.191 | 0.847 | 0.578 | 0.947 |
|  | B.I. 3 genes (r) | 0.907 | 0.567 | 0.579 | -0.314 | 0.29 | -0.371 | 0.757 | 0.718 | 0.116 | 0.746 | 0.54 | 0.842 |
|  | B.I. 2 genes (r) | 0.911 | 0.175 | 0.682 | -0.495 | 0.063 | -0.503 | 0.869 | 0.803 | 0.146 | 0.907 | 0.74 | 0.889 |
|  | overall B. I.* (r) | 0.891 | 0.419 | 0.665 | -0.421 | 0.168 | -0.430 | 0.815 | 0.765 | 0.181 | 0.824 | 0.619 | 0.893 |
|  | S.D. | 0.274 | 0.418 | 0.671 | 2.732 | 0.525 | 0.481 | 0.830 | 0.727 | 0.671 | 0.348 | 0.557 | 0.512 |
| GeNorm | M | 0.295 | 0.545 | 0.446 | 1.043 | 0.606 | 0.654 | 0.431 | 0.412 | 0.498 | 0.258 | 0.368 | 0.283 |
| NormFinder | stability | 0.093 | 0.105 | 0.435 | 2.061 | 0.25 | 0.343 | 0.535 | 0.461 | 0.512 | 0.054 | 0.354 | 0.211 |

Abbreviations: S.D. = Standard deviation, B.I. = BestKeeper Index, *r* = Pearson product-moment correlation coefficient.

*average B.I.

**Table S2F. Descriptive statistical analysis of the candidate reference gene expression in whole charr embryos using** three algorithms.

| **Algorithm** |  | **ACTB** | **b2m** | **EF1a** | **GAPDH** | **HPRT** | **IF5A1** | **RL7** | **RS9** | **RS20** | **TBA** | **UB2L3** | **UBIQ** |
| --- | --- | --- | --- | --- | --- | --- | --- | --- | --- | --- | --- | --- | --- |
| BestKeeper | B.I. all (r) | 0.608 | 0.696 | 0.843 | -0.196 | 0.801 | 0.144 | 0.427 | 0.733 | 0.806 | 0.446 | 0.799 | 0.497 |
|  | B.I. 4 genes (r) | 0.542 | 0.479 | 0.938 | -0.527 | 0.836 | -0.151 | 0.596 | 0.889 | 0.813 | 0.598 | 0.844 | 0.654 |
|  | B.I. 3 genes (r) | 0.512 | 0.593 | 0.904 | -0.424 | 0.803 | -0.155 | 0.549 | 0.905 | 0.832 | 0.446 | 0.893 | 0.491 |
|  | B.I. 2 genes (r) | 0.639 | 0.668 | 0.9 | -0.34 | 0.808 | -0.035 | 0.491 | 0.802 | 0.799 | 0.442 | 0.825 | 0.477 |
|  | overall B. I.* (r) | 0.575 | 0.609 | 0.896 | -0.372 | 0.812 | -0.049 | 0.516 | 0.832 | 0.813 | 0.483 | 0.840 | 0.530 |
|  | S.D. | 0.353 | 1.094 | 0.421 | 1.292 | 0.496 | 0.580 | 0.568 | 0.325 | 0.567 | 0.516 | 0.289 | 0.534 |
| GeNorm | M | 0.403 | 0.613 | 0.288 | 0.761 | 0.326 | 0.509 | 0.439 | 0.247 | 0.272 | 0.379 | 0.353 | 0.223 |
| NormFinder | stability | 0.172 | 0.664 | 0.174 | 0.993 | 0.224 | 0.406 | 0.399 | 0.148 | 0.276 | 0.36 | 0.204 | 0.254 |

Abbreviations: S.D. = Standard deviation, B.I. = BestKeeper Index, *r* = Pearson product-moment correlation coefficient.

*average B.I.

**Table S3. Ranking of the candidate reference genes in the heads or the different Arctic charr groups using NormFinder (Nf) and Standard deviation (SD)**

| **Gene** | **AC heads** | | **PL heads** | | **SB heads** | | **LB heads** | |
| --- | --- | --- | --- | --- | --- | --- | --- | --- |
|  | **Nf** | **SD** | **Nf** | **SD** | **Nf** | **SD** | **Nf** | **SD** |
| ACTB | 1 | 4 | 2 | 4 | 1 | 2 | 2 | 1 |
| b2m | 11 | 11 | 7 | 11 | 4 | 1 | 3 | 3 |
| EF1α | 5 | 3 | 8 | 6 | 6 | 7 | 8 | 8 |
| HPRT | 7 | 9 | 4 | 5 | 5 | 5 | 5 | 6 |
| IF5A1 | 6 | 6 | 10 | 3 | 9 | 6 | 6 | 4 |
| RL7 | 10 | 7 | 11 | 8 | 10 | 10 | 11 | 11 |
| RS9 | 9 | 7 | 9 | 10 | 11 | 11 | 9 | 10 |
| RS20 | 8 | 10 | 6 | 7 | 7 | 8 | 10 | 9 |
| TBA | 2 | 1 | 1 | 1 | 3 | 3 | 1 | 2 |
| UB2L3 | 3 | 2 | 3 | 2 | 8 | 9 | 7 | 7 |
| UBIQ | 4 | 5 | 5 | 9 | 2 | 4 | 4 | 5 |

Abbreviations: AC = aquaculture, PL = planktivorous, LB = large benthic, SB = small benthic/dwarf
