# Supplementary material for: Validation of Reference Genes for Expression Studies during Craniofacial Development in Arctic Charr
Source: PLoS One. 2013 Jun 13;8(6):e66389. doi: 10.1371/journal.pone.0066389 (PMC3681766; doi:10.1371/journal.pone.0066389)
Supplement: File S2 — Contains: Figure S1 Comparison of expression levels of the eleven candidate reference genes in heads and whole embryos using qPCR. The genes are ranked from left to right as most to least differentially expressed between whole embryos (whole) and head (corresponding P-values are shown below the x-axis). Insert in A displays samples (black filled spots) used in both analyses. (A) Boxplot shows the range of Cq values for each candidate reference gene in whole embryo (white) and head (gray) homogenates. Displayed are the median, the 25th and 75th percentiles and the minimum and maximum Cq values for each gene. (B) Relative quantity for each reference gene candidate in whole embryos (open circles) and head (grey diamond) homogenates. The whiskers represent ±0.95 confidence interval of the mean. Figure S2 Optimal number of reference genes for normalisation. GenormPLUS was used to determine the optimal number of reference genes in head and whole embryo homogenates. LB: large benthic; SB: small benthic/dwarf; PL: planktivorous; AC: aquaculture. Average pair-wise variations (Vn/n+1) were calculated using the genes ranked according to GeNorm. The recommended cut-off value of 0.15 is shown by a dashed line and below this line the benefit of using an extra reference gene is limited. (DOCX) [file pone.0066389.s002.docx]

File S2

Figure S1

**A**

**B**

Figure S2
